# Global cluster analysis and network visualization in organoids in cancer research: a scientometric mapping from 1991 to 2021

**DOI:** 10.3389/fonc.2023.1253573

**Published:** 2023-09-15

**Authors:** Shunshun Tan, Jiali Deng, Haobin Deng, Lijun Lu, Zhenzhe Qin, Yu Liu, Lifeng Tang, Zhonghua Li

**Affiliations:** Department of Oncology, Liuzhou People’s Hospital Affiliated to Guangxi Medical University, Liuzhou, China

**Keywords:** global research trend, organoid, cancer, bibliometrics, visualized study

## Abstract

**Objective:**

In the last three decades, there has been a surge in research on cancer organoids using 3D culture technologies, which has resulted in the development of physiological human cancer models. This study aims to provide an overview of the global trends and frontiers in research on cancer organoids.

**Methods:**

A total of 3189 publications on organoids in cancer research from 1991 to 2021 were collected from the Science Citation Index-Expanded (SCIE) of Web of Science (WoS). Bibliometric methods such as the R package “Bibliometrix,” Citespace, and VOS viewer software were employed to investigate and visualize bibliographic coupling, co-citation, co-authorship, and co-occurrence trends, as well as publication trends in the field of organoids in cancer research.

**Results:**

From 1991 to 2021, there has been a significant increase in publications on cancer organoids, with most articles being from North America, Eastern Asia, and Western Europe. The USA had the highest number of publications, citations, prolific authors, and research funding globally. Cancers was the journal with the most publications, while Nature had the best total link strength. Harvard University were the most contributive institutions. The global research in this field could be classified into five clusters: chemotherapy study, organoids for drug screening, different models, molecular mechanism study, and organoid construction. These areas are expected to remain hotspots for future research.

**Conclusions:**

The number of publications on organoids in cancer research is expected to increase based on current global trends.

## Introduction

In the context of global population aging, the prevalence of cancer is expected to continue increasing, posing a significant health burden worldwide (1–3). Over the past few decades, cancer treatment has progressed with the adoption of surgical excision followed by chemoradiotherapy, leading to improved patient survival rates. Furthermore, preventive measures and targeted therapies hold promise in reducing cancer-related deaths. However, a major challenge in translating scientific knowledge from the laboratory to clinical practice lies in the limited ability of many cancer models to accurately represent the heterogeneity observed in actual cancer patients (4). Traditional cancer models can be classified into two-dimensional (2D) cell line cultures, patient-derived tumor xenografts (PDTXs), and genetically engineered mouse models. Nevertheless, the clinical application of 2D cell line cultures is hindered by several drawbacks, including their inability to replicate the complex tumor immune microenvironment and organ-specific functions, as well as the loss of genetic heterogeneity after multiple passages (5, 6). Animal models, on the other hand, have significantly contributed to our understanding of the fundamental aspects of cancer. However, the process of establishing animal cancer models is often time-consuming, and some models fail to completely mimic the pathogenic processes observed in human patients, making it challenging to design successful clinical trials (54). Therefore, the advancement of personalized cancer therapies necessitates the development of novel cancer models that can overcome the limitations of current approaches.

Organoids are a type of model where adult stem cells derived from tissues are embedded in a three-dimensional matrix, allowing them to efficiently self-organize into structures resembling organs (7). These organoids possess attractive features such as multiple cell types mirroring their in vivo counterparts, a cellular organization similar to primary tissues, and organ-specific functions. As a result, organoids have emerged as valuable models for studying and developing patient-specific cancer treatments (8). In recent years, organoids have found wide application as preclinical models in cancer research, including personalized cancer treatments (9), drug development (10), and investigating tumorigenesis mechanisms (11), among others. Since the pioneering work by Sato et al. in 2009, where they successfully generated three-dimensional epithelial organoids from single leucine-rich repeat-containing G protein-coupled receptor 5 (LGR5) + intestinal stem cells (12), significant technological advancements have been made in organoid culture protocols. Epithelial cancer organoid cultures have expanded to various branches, encompassing colon (13), liver (14), pancreas (15), prostate (16), stomach (17), lung (18), breast (19), and other organs. However, many of these areas are still in the early stages of exploration, and there remain several challenges to overcome for the widespread application of organoids in cancer research. Firstly, organoids inherently lack the presence of stroma, blood vessels, and immune cell types, which are crucial components of the tumor microenvironment. This limitation restricts the ability of organoids to fully recapitulate the complex interactions between cancer cells and their surrounding environment. Secondly, the reliance on Matrigel or other mouse-derived extracellular matrix substitutes in organoid culture may introduce variables that could influence experimental outcomes. Moreover, studies on non-epithelial cancers using organoids are limited, and the regulatory mechanisms underlying the influence of the extracellular matrix in driving the phenotype and drug resistance of cancer organoids remain not fully understood.

Despite the challenges, cancer organoids have emerged as a valuable high-throughput platform for studying cancer in vitro and developing personalized anti-cancer treatments, including chemotherapies (19), immunotherapies (20), and radiation therapies (21). Recent reviews have provided comprehensive summaries of the current advancements and future prospects of organoids in cancer research. However, the presence of scattered keywords in these articles makes it difficult to precisely locate information across various databases. Therefore, conducting a quantitative and qualitative analysis of the global research on organoids in cancer research could provide valuable reference guidelines for future researchers. This analysis would involve examining numerical growth trends, identifying the contributions of countries, institutions, and authors, and predicting promising hot topics to guide and direct research efforts in this field.

Drawing upon literature metrology characteristics and databases, we have employed a bibliometric analysis to explore qualitative and quantitative information, enabling us to assess the trends of research activities over time. Bibliometric analysis, a quantitative method used in library and information science, involves analyzing bibliographic material to predict the development of a particular field (22–25). These viable methods have been successfully applied in evaluating research trends in various domains, including cancer gene therapy (26), orthopedics (27), and the microbiome (28), among others (29, 30). However, there has been a lack of adequate investigation into the global development trends of organoids in cancer research. To bridge this gap, we conducted a comprehensive search of studies conducted between 1991 and 2021 in the field of organoids in cancer research. Subsequently, we performed a bibliometric analysis to evaluate the current status and global trends in this area

## Materials and methods

### Data source and search strategies

For our research, we utilized the Science Citation Index Expanded (SCIE) database from Web of Science (WoS). This database comprises over 12,000 international academic journals and is widely regarded as one of the most comprehensive and authoritative platforms for obtaining global academic information (31, 32). To ensure the reliability and inclusiveness of our study, we conducted a search within the WoS database, which was up to date as of a single day, July 1, 2022, to avoid any potential bias caused by rapid database updates.

The search strategy employed was as follows: we searched for studies related to organoids in cancer research and performed a bibliometric analysis, building upon previous studies (26, 33). The search criteria included the following parameters: the theme was set as “Organoid” or “Organoids,” combined with themes such as “cancer,” “tumor,” “neoplasm,” “carcinoma,” or “phyla.” We limited the publishing year range to 1991-2021 and restricted the language to English. Additionally, we specified the document types as “Article” or “Review” to focus on peer-reviewed publications.

To ensure accuracy in assessing the contributions of specific countries or regions, we refined the detailed information by indexing the country/region field in the WoS database. In terms of inclusion criteria, we considered peer-reviewed publications primarily focused on the research field of organoids in cancer research. The document types had to be limited to “Article” and “Review,” and the publications had to be written in English. Furthermore, we only included publications with publishing dates between 1991 and 2021. Exclusion criteria were applied as well, which included publications not related to organoids in cancer research and documents such as news articles, meeting abstracts, briefings, and other similar types of publications.

### Data collection and cleaning

**Figure 1:**
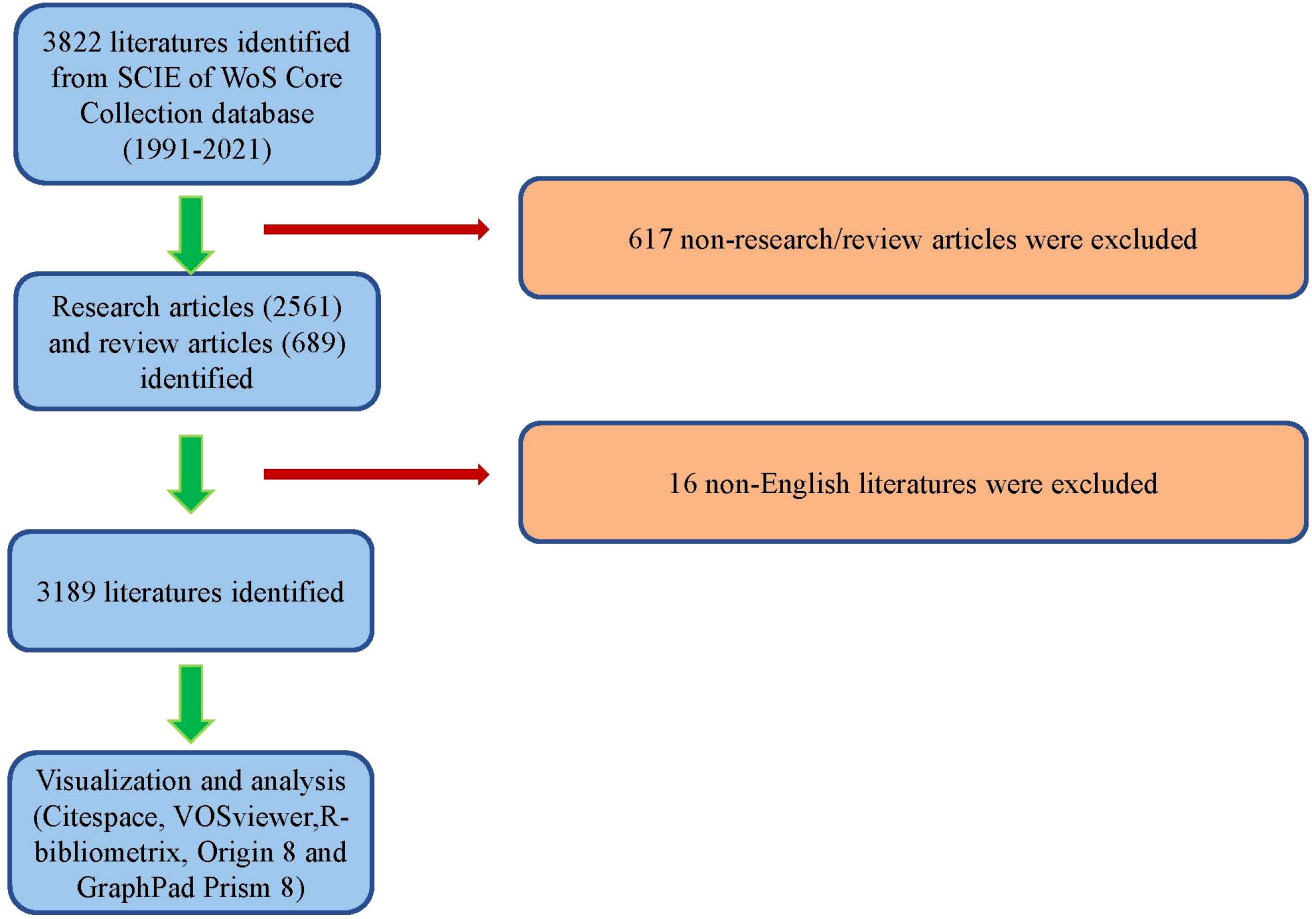
Flow diagram of the study identification and inclusion process.

To initiate the screening process for our research, we extracted all relevant information from the publications in the WoS database, including the publication year, title, authors’ names, affiliations, nationalities, abstracts, keywords, and journal names. This data was saved as.txt files and subsequently imported into Excel 2021 for further analysis. Two co-authors, designated as SSY and JLD, conducted a manual review of the publications to identify and eliminate any articles that were deemed irrelevant to our study. Any discrepancies that arose during this review process were discussed among the team, and a consensus was reached regarding whether to include or exclude the articles from our study. Lastly, all authors independently performed data cleaning procedures, following the process outlined in **Figure 1**.

### Bibliometric analysis

The selection of the WoS database for our bibliometric research was based on its comprehensive coverage and its ability to provide essential characteristics of eligible publications. To conduct the bibliometric analysis, we utilized software such as Origin 8 and GraphPad Prism 8. These software packages allowed us to plot the number of publications against the year of publication, providing insights into the trend of publications over time. The year of publication was represented on the x-axis, while the number of publications was represented on the y-axis. In addition to examining publication trends, we calculated the relative research interest (RRI). RRI is a measure of the number of publications in a specific field divided by the total number of publications in all fields per year. This calculation helps to gauge the level of research interest in the field of organoids in cancer research. To assess the academic productivity and influence of researchers or countries, we utilized the H-index. The H-index is a crucial indicator in evaluating the quality of articles and reflects the number of publications and corresponding citations received by researchers or countries (34). To visualize the geographical distribution of publications, we employed R software, which incorporates various libraries such as python, numpy, scipy, and matplotlib (35). This allowed us to generate maps and graphical representations of the publication distribution across different regions and countries.

### Visualized analysis

In this study, we used Bibliometrix to visualize the international collaboration between countries. The parameters used for this analysis were set as follows: minimum edges of 2 and an edge size of 5, which helped in highlighting significant collaborations between countries. Furthermore, we employed Citespace (version 6.1. R2) to enable the visualization of the dual-map overlay, which illustrated the connections between journals in terms of shared references. To further analyze co-authorship, co-citation, and co-occurrence patterns, we constructed bibliometric maps using VOS viewer (version 1.6.14, Leiden University, Leiden, The Netherlands). VOS viewer facilitated the construction and visualization of bibliometric networks, which provided a more comprehensive understanding of the relationships between authors, references, and keywords. We utilized VOS viewer for bibliographic coupling analysis, co-citation analysis, co-authorship analysis, and co-occurrence analysis. The parameters for these analyses were determined based on specific criteria. For example: 1) For bibliographic coupling analysis, the minimum number of documents required for analysis was set as more than 20 for Country, more than 20 for Journal, and 10, 40 for Author and Institution. 2) For co-citation analysis, the minimum number of citations required for analysis was set as more than 200, 100, and 500 for Author, Reference, and Journal. 3) For co-authorship analysis, the minimum number of documents required for analysis was set as more than 20, 10, and 30 for Country, Author, and Institution. 4) For co-occurrence analysis, keywords were considered if they appeared more than 50 times in titles or abstracts across all papers. These parameter settings helped ensure that the analyses focused on significant patterns and relationships within the dataset, enhancing the validity and relevance of the results obtained.

## Results

### Global contribution to the field

**Figure 2:**
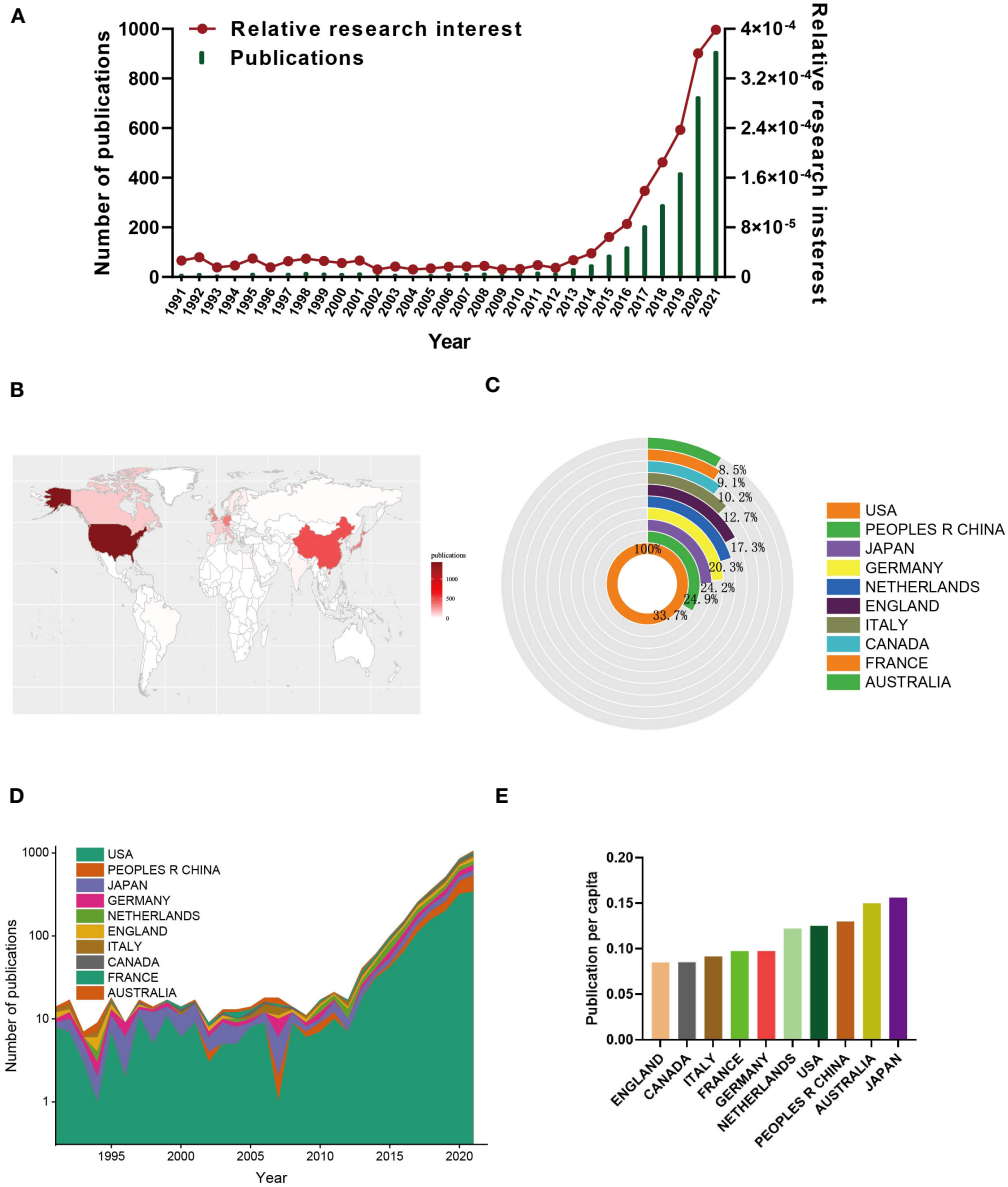
Global trends and countries/regions contributing to the research field regarding organoids in cancer research. **(A)** The annual number of publications and relative research interest (RRI) related to organoids in cancer research. **(B)** A world map depicting distribution of organoids in cancer research. **(C)** The sum of organoids in cancer research related publications in the top 10 countries/regions. **(D)** The annual number of publications in the top 10 most productive countries from 1991 to 2021. **(E)** The publication per capita in top 10 countries from1991 to 2021.

Following the literature screening process, a total of 3,189 eligible publications were included in the final analysis, as shown in **Figure 1**. The number of annual publications displayed an upward trend from 1991 to 2021, reaching its peak of 911 publications in 2021. The year 2021 accounted for the highest percentage of research articles, representing 28.57% of the total number (**Figure 2A**). In addition, the relative interest in this field has also increased over the past few years.

A total of 73 countries and regions contributed to the publications in this field. The United States ranked first with 1,429 papers (44.81%), followed by China (481, 15.08%), Japan (356, 11.16%), Germany (346, 10.85%), and the Netherlands (290, 9.09%) (**Figures 2B, C**). Notably, China’s publication growth has shown rapid acceleration since 2015 (**Figure 2D**).

Furthermore, the top ten countries/regions with the strongest citation bursts (indicating a significant surge in publications within a short period) (**Figure 2D**) revealed that Japan had the highest burst strength (15.53) from 1991 to 2008, indicating a substantial number of scholars studying cancer organoids in Japan during this period. And researchers in Japan also presented with highest publication per capita among top 10 countries (**Figure 2E**). However, many other countries also demonstrated citation bursts, suggesting the continuous growth of this research field.

**Figure 3:**
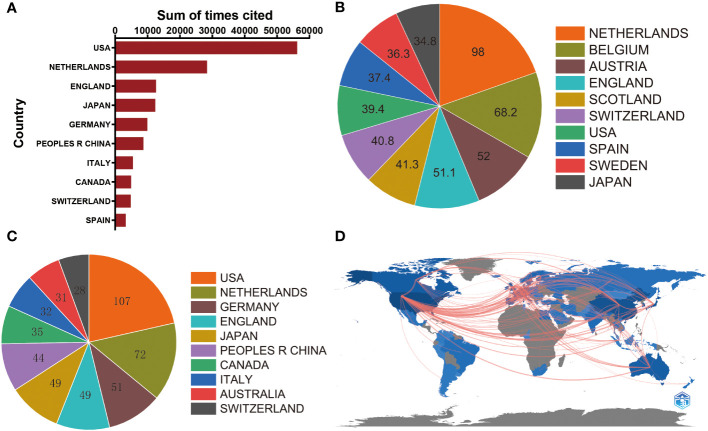
**(A)** The top 10 countries/regions of total citations of organoids in cancer research publications. **(B)** The top 10 countries/regions of the average citations per paper of organoids in cancer research publications. **(C)** The top 10 countries/regions of the H-index of organoids in cancer research publications. **(D)** The geographical network map of organoids in cancer research publications.

In terms of total citation frequency, the top five countries with the highest citation frequencies were the United States (56,238), Netherlands (28,418), England (12,627), Japan (12,396), and Germany (9,940) (**Figure 3A**). The Netherlands (98) ranked first in terms of average citation frequencies, followed by Belgium (68.2), Austria (52), England (51.1), and Scotland (41.3) (**Figure 3B**). The United States had the highest H-index (107) among the countries, followed by the Netherlands (72), Germany (51), England (49), and Japan (49) (**Figure 3C**). Finally, the cooperation network diagram mainly showed collaborations within North America, Western Europe, and East Asia (**Figure 3D**).

### Analysis of global publications

#### Authors

**Figure 4:**
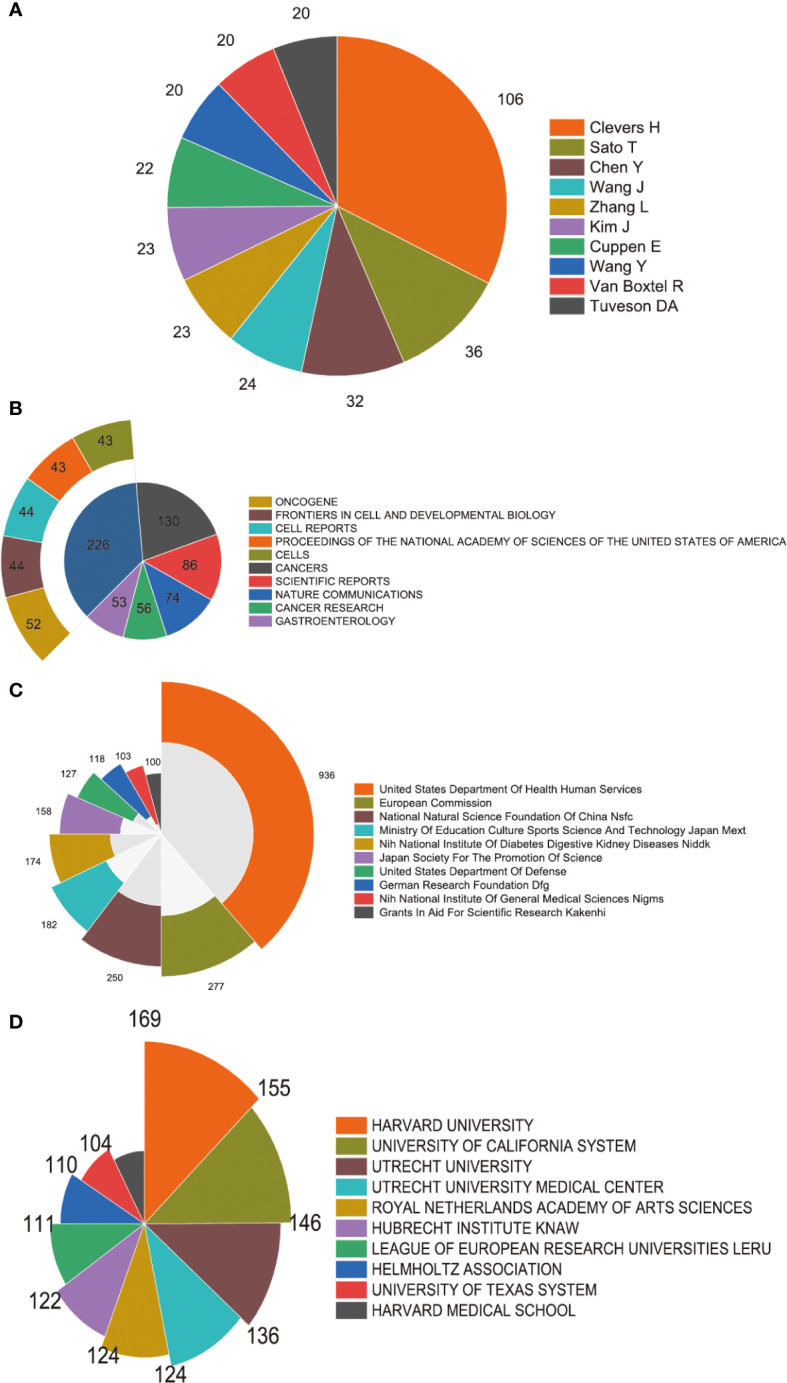
High-contribution authors, journals, institutions and funds of global publications about organoids in cancer research. **(A)** The top 10 authors with most publications on the organoids in cancer research. **(B)** The top 10 journals with most publications on the organoids in cancer research. **(C)** The top 10 institutions with most publications on the organoids in cancer research. **(D)** The top 10 funding sources with most publications on the organoids in cancer research.

Moreover, our analysis delved into the distribution of authorship in the field of cancer organoids. The findings revealed that a considerable proportion of publications were contributed by the top 10 authors. Specifically, these authors collectively published 326 papers, which accounted for 18.47% of all publications in this field. Notably, Hans Clevers emerged as the most prolific author with 106 studies on cancer organoids, signifying his significant contributions to the field. Toshiro Sato followed closely with 36 publications, while Yu Chen had 32 publications (**Figure 4A**). The substantial number of publications by these authors reflects their profound impact on the field of cancer organoids, highlighting their extensive expertise and experience. Their research has advanced our knowledge of cancer biology and paved the way for innovative therapeutic approaches.

#### Journal analysis

In our study, we conducted a comprehensive analysis of journal distribution to identify the active journals in various fields related to cancer organoids. The top 10 journals with the highest number of publications are presented in **Figure 4B**. Notably, Cancers (impact factor = 5.2, 2022) ranked the highest with 130 publications. Scientific Reports (IF = 4.6, 2022) followed closely with 86 publications, while Nature Communications (IF = 16.6, 2022) had 74 publications. Cancer Research (IF = 11.2, 2022) and Gastroenterology (IF = 29.4, 2022) also made significant contributions with 56 and 53 publications, respectively. Besides these top 5 journals, other prominent journals in the field include Cell Stem Cell (IF = 23.9, 2022) with 46 articles, Oncotarget (IF =NA, 2022) with 39 articles, Stem Cell Reports (IF = 5.9, 2022) with 37 articles, and Lab on a Chip (IF = 6.1, 2022) with 32 articles. These journals cover a wide range of research areas, including cancer research, stem cell research, microfluidics, and lab-on-a-chip technology.

In terms of geographical distribution, the top 5 journals were primarily affiliated with the United States, with 18 journals based in the country. England and Switzerland had 2 journals each, while Canada and Germany had 1 journal each among the top 25. This distribution highlights the leading role of the United States in publishing research on cancer organoids. It is worth noting that a significant number of journals in the top 25 are open access, indicating the increasing prominence of open access publishing in this field. This observation underscores the importance of open access as a publishing model in facilitating the dissemination of research findings. The analysis of journal distribution provides valuable insights for researchers to select suitable journals for their publications and helps identify leading journals in the field of cancer organoids.

#### Institution output

**Figure 4C** showcases the top 10 institutions based on their number of publications in the field of cancer organoids. Harvard University secures the first position with 169 publications, highlighting its significant contributions to this area of research. The University of California System follows closely with 155 publications, indicating its strong presence in the field. Utrecht University ranks third with 146 publications, demonstrating its substantial research output in cancer organoids. These institutions have played a crucial role in advancing the understanding of cancer biology and the development of organoid-based research approaches.

#### Funding sources

In **Figure 4D**, the distribution of the top 10 funding sources is presented. The United States Department of Health Human Services secures the first place, providing funding for 936 publications in the field of cancer organoids. The European Commission takes the second place, supporting 277 publications through its funding programs. The National Natural Science Foundation of China (NSFC) ranks third, with funding support for 250 publications. These funding sources have played a crucial role in supporting research endeavors and driving advancements in the field of cancer organoids, enabling scientists to investigate new avenues of study and develop innovative approaches for cancer treatment and therapeutics.

#### Caner types

In **Table 1**, the distribution of the common studied cancer types with the most references is presented. The Colon and Rectal Cancer secures the first place, providing for 611 publications in the field of cancer organoids. The Breast Cancer takes the second place, supporting 417 publications. The Liver Cancer ranks third, with support for 314 publications. These different types of cancer provide a basic overview of the types that currently pose major threats to human health, and guide researchers and clinicians in the direction of research efforts and innovative therapies to overcome these cancer types.

**Table 1:** The common studied cancer types with the most references on organoids in cancer research.

Rank	Can types	Total references
1	Colon and Rectal Cancer	611
2	Breast Cancer	417
3	Liver Cancer	314
4	Lung Cancer	278
5	Pancreatic Cancer	261
6	Kidney Cancer	219
7	Prostate Cancer	204
8	Cerebral Cancer	199
9	Gastric Cancer	173
10	Bladder Cancer	59
11	Leukemia	52
12	Melanoma	41
13	Endometrial Cancer	34
14	Non-Hodgkin Lymphoma	29
15	Esophagus Cancer	28
16	Thyroid Cancer	26
17	Leukemia	52
18	Others	244

### Bibliographic coupling analysis

#### Country

**Figure 5:**
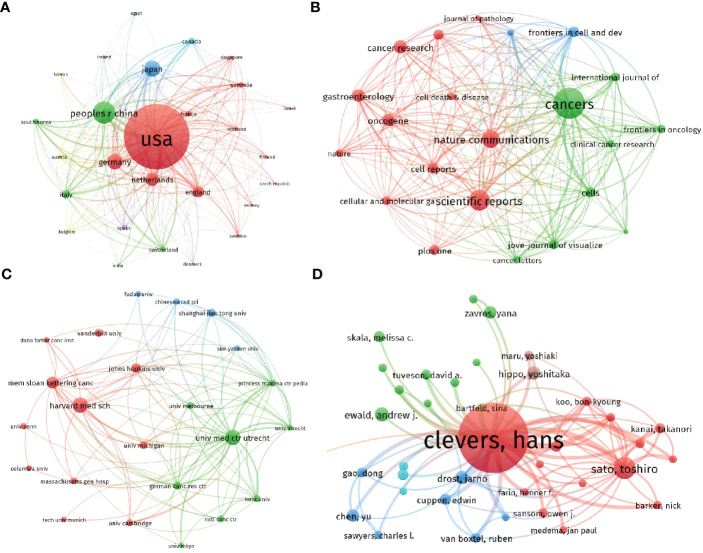
Mapping of bibliographic coupling analysis regarding the organoids in cancer research. **(A)** Mapping of the countries on the organoids in cancer research. **(B)** Mapping of the identified journals on organoids in cancer research. **(C)** Mapping of the institutions on the organoids in cancer research. **(D)** Mapping of the authors on organoids in cancer research. The thickness of the line represents the link strength between the journals/institutions/countries/authors.

In **Figure 5A**, a collaboration network map generated using VOS viewer is presented to analyze the collaboration between countries in the field of cancer organoids. The map consists of 27 nodes representing different countries, with the size of each node indicating the total number of publications contributed by that country. The color of the nodes represents the clusters of countries that exhibit similar collaboration patterns. The map reveals three main clusters: North America, Europe, and Asia. The North American cluster is the most prominent, with the United States being the most connected country in the network. The European cluster consists mainly of countries in Western Europe, while the Asian cluster includes countries from East and Southeast Asia. This collaboration network highlights the active engagement of researchers and institutions from these regions in advancing the field of cancer organoids. The map provides a visual representation of the collaborative relationships between countries and offers insights into the global landscape of research in this field. It demonstrates the importance of international collaboration in driving scientific progress and knowledge sharing in the domain of cancer organoids.

#### Journal

Bibliographic coupling analysis was conducted to assess the similarity between documents, and the results were visualized using VOS viewer. For this analysis, a minimum number of documents set as 20 was used to identify the journals with significant link strength. **Figure 5B** presents the results, with a total of 23 journals identified. The top 5 journals with the highest total link strength are as follows: Cancers (impact factor = 6.575, 2021, total link strength = 101301); Nature Communications (IF = 17.694, 2021, total link strength = 54496); Scientific Reports (IF = 4.996, 2021, total link strength = 45616); Frontiers in Cell and Developmental Biology (IF = 6.081, 2021, total link strength = 44102); Cells (IF = 7.666, 2021, total link strength = 39909). These journals have exhibited a high degree of bibliographic coupling, indicating that they have published a significant number of articles that are interconnected and share similar references. This finding suggests that these journals are central to the dissemination and exchange of knowledge in the field of cancer organoids. Researchers can refer to these journals to access a comprehensive collection of relevant literature and stay updated on the latest developments and research trends.

#### Institution

An analysis of papers from 458 institutions was performed using VOS viewer, and the results are presented in **Figure 5C**. The analysis considered papers that were associated with a minimum of 40 documents used by an institution and a maximum of 23 organizations per document. The top 5 institutions with the highest total link strength, indicating strong connections and collaborations with other institutions, are as follows: University Medical Center Utrecht (total link strength = 586,513); Princess Máxima Center for Pediatric Oncology (total link strength = 314,899); Harvard Medical School (total link strength = 291,079) German Cancer Research Center (total link strength = 285,148); Memorial Sloan Kettering Cancer Center (total link strength = 284,662). These institutions have demonstrated significant collaboration and engagement within the field of cancer organoids, as evidenced by their high total link strengths. The findings highlight the importance of these institutions in advancing research and knowledge in the area of cancer organoids. Researchers can consider these institutions as valuable sources of expertise and potential collaborative opportunities for furthering their own research in this field.

#### Author

An analysis of publications contributed by 328 authors was conducted using VOS viewer, and the results are depicted in **Figure 5D**. The analysis considered authors with a minimum number of documents greater than 5. The top 5 most productive authors in terms of total link strength, which represents their collaboration and co-authorship relationships with other authors, are as follows: Hans Clevers (total link strength = 278,090); Toshiro Sato (total link strength = 80,326); Jarno Drost (total link strength = 55,958); Bon-Kyoung Koo (total link strength = 46,705); Daniel E. Stange (total link strength = 44,559). These authors have made significant contributions to the field of cancer organoids, as evidenced by their high total link strengths. Their extensive collaborations with other researchers highlight their expertise and influence in this area of research. Researchers can consider these authors as key figures in the field and explore their work for valuable insights and potential collaboration opportunities.

### Co-citation analysis

#### Authors

**Figure 6:**
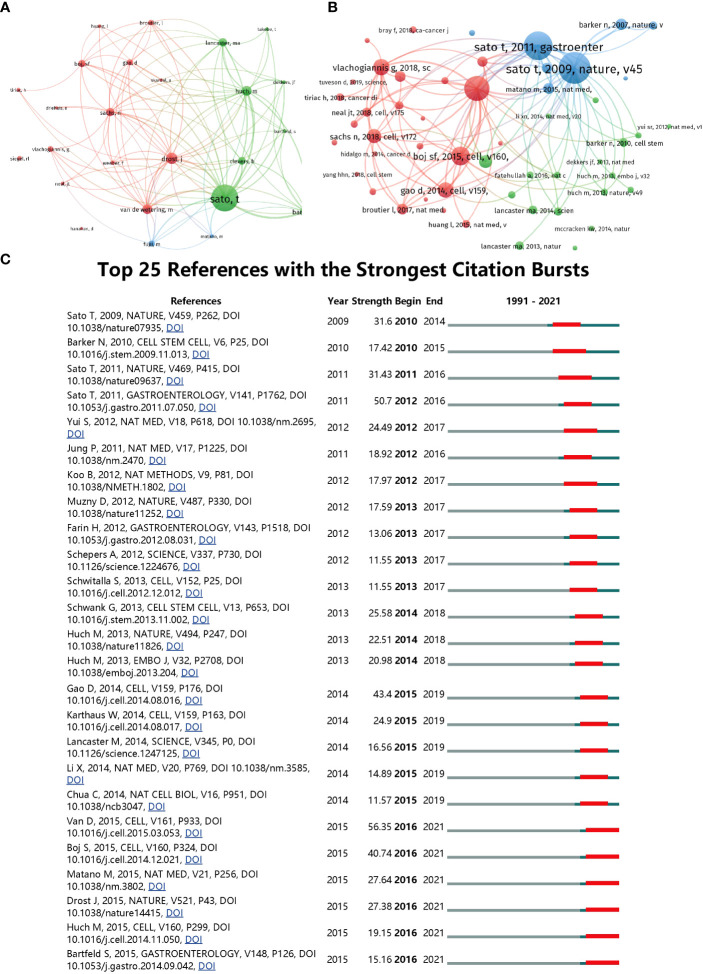
**(A)** Mapping of the co-cited authors related to this field. (The points with different colors represent the identified authors. **(B)** Mapping of the co-cited references related to this field. (The points with different colors represent the cited references.) The size of burble represents the citation frequency. The shorter the line, the closer the link between two papers. The same color of the points represents the same research area they belong to. **(C)** Top 25 references with strongest citation bursts of publications related to organoids in cancer research.

In the co-citation analysis using VOS viewer, 25 authors with a minimum of 200 citations were analyzed, as shown in **Figure 6A**. Co-citation analysis measures the relatedness between items based on the number of times they were co-cited. The top 5 publications with the largest total link strength in this analysis were as follows: Sato, T (total link strength = 47042); Drost, J (total link strength = 26597); Huch, M (total link strength = 24937); Barker, N (total link strength = 21772); Lancaster, Ma (total link strength = 19781). Additionally, the top 10 authors with the most citations were identified and listed in **Table 2**. Sato, T had the highest total citations with 1660, followed by Drost, J (total citation: 704), Barker, N (total citation: 692), Huch, M (total citation: 607), and Van De Wetering, M (total citation: 601). It is worth noting that half of these authors are from the Netherlands, indicating their leading position in terms of total citation count compared to authors from other countries.

**Table 2:** The top 10 authors with the most citations on organoids in cancer research.

Rank	Co-cited Authors	Country	Total citations
1	Sato, T	Japan	1660
2	Drost, J	Netherlands	704
3	Barker, N	Singapore	692
4	Huch, M	England	607
5	Van De Wetering, M	Netherlands	601
6	Sachs, N	Netherlands	517
7	Lancaster, Ma	USA	513
8	Clevers, H	Netherlands	482
9	Boj, Sf	Netherlands	447
10	Fujii, M	Japan	380

#### Reference

In the reference co-citation analysis using VOS viewer, 58 references with a minimum of 100 documents citing them were analyzed. This analysis helps uncover the relationship between items based on their total citation and track the advancement of a research area. The top 5 articles with the highest total link strength in this analysis were:

(12), Nature, v459, p262 (total link strength = 14541); (36), Cell, v161, p933 (total link strength = 13343); (13), Gastroenterology, v141, p1762 (total link strength = 13100); (15), Cell, v160, p324 (total link strength = 9974); (37), Cell, v159, p176 (total link strength = 8621). Furthermore, using CiteSpace, the top 25 references with the strongest citation bursts were identified, as presented in **Figure 6C**. The article titled “Prospective derivation of a living organoid biobank of colorectal cancer patients,” published in 2015, ranked first with a burst strength of 56.35.

**Table 3** presents the top 10 co-cited references, where Sato T, the first author, contributed the top two co-cited articles: “Single Lgr5 stem cells build crypt-villus structures in vitro without a mesenchymal niche” (38) with a total citation of 700, and “Long-term expansion of epithelial organoids from human colon, adenoma, adenocarcinoma, and Barrett’s epithelium” (39) with a total citation of 630. It is also noteworthy that the corresponding author Clevers H accounted for 7 out of the top 10 co-cited references.

**Table 3:** The top 10 co-cited journals related to organoids in cancer research.

Rank	Cited Journal	Citations	IF (2021)
1	*Nature*	10165	64.8
2	*Cell*	8096	64.5
3	*P Natl Acad Sci USA*	5576	11.1
4	*Cancer Research*	4932	11.2
5	*Science*	4616	56.9
6	*Cell Stem Cell*	3512	23.9
7	*Nature Medicine*	3452	82.9
8	*Gastroenterology*	3302	29.4
9	*Plos One*	3183	3.7
10	*Nature Communication*	3056	16.6

### Analysis of journals and research areas

**Figure 7:**
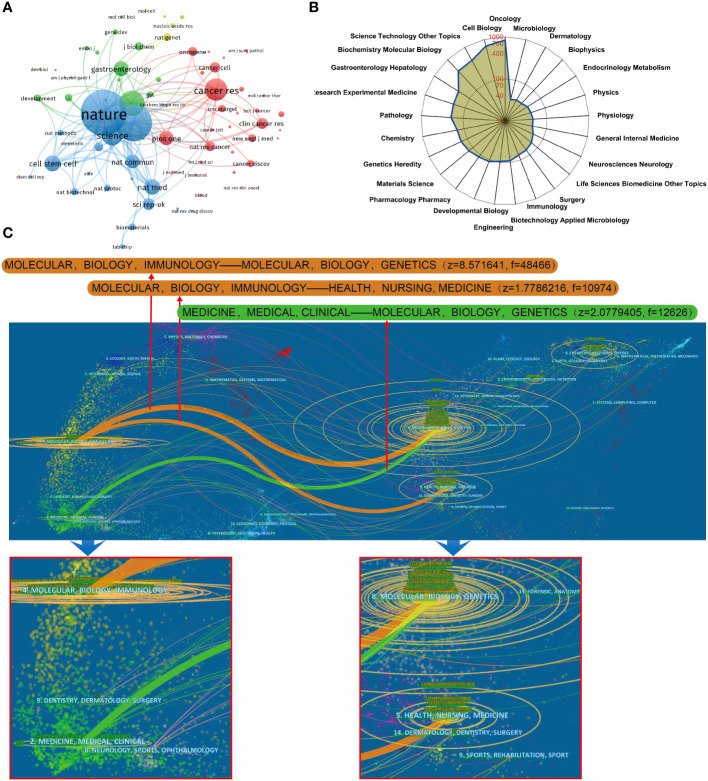
**(A)** Mapping of the co-cited journals related to this field. (The points with different colors represent the identified journals.) **(B)** The top 25 research orientations with most publications on the organoids in cancer research. **(C)** The dual-map overlay of journals related to the organoids in cancer research.

Co-citation analysis of journals is a valuable method to understand the interrelatedness of journals within a specific research field. In this study, 72 journals with a minimum of 500 citations were analyzed using VOS viewer. The findings are as follows:

**Figure 7A** displays the top 5 journals with the highest total link strength: Nature, Cell, P Natl Acad Sci USA, Cancer Research, and Science. These journals are widely recognized as leading journals in the field of cancer research and have published numerous significant papers related to organoids. Among them, Nature has the highest total citations with 10,165. **Figure 7B** illustrates the top 25 research orientations related to organoids in cancer research. The most popular research fields include oncology, cell biology, science technology other topics, biochemistry molecular biology, and gastroenterology hepatology. This indicates that organoid research is a multidisciplinary field that attracts researchers from various areas of expertise. Furthermore, a dual-map overlay of journals related to organoids in cancer research was performed, as shown in **Figure 7C**. The colored path represents the citation association, and the spline wave from left to right describes the association. Three primary citation paths marked in orange and green were identified. The first two paths indicate that documents published in molecular/biology/genetics journals are primarily cited by researchers published in molecular/biology/immunology and medicine/medical/clinical journals. The third path suggests that documents published in health/nursing/medicine journals are primarily cited by researchers published in molecular/biology/immunology journals. This analysis provides insight into the interdisciplinary nature of organoid research in cancer and highlights the importance of collaborations between different fields of research.

### Co-authorship analysis

#### Countries

**Figure 8:**
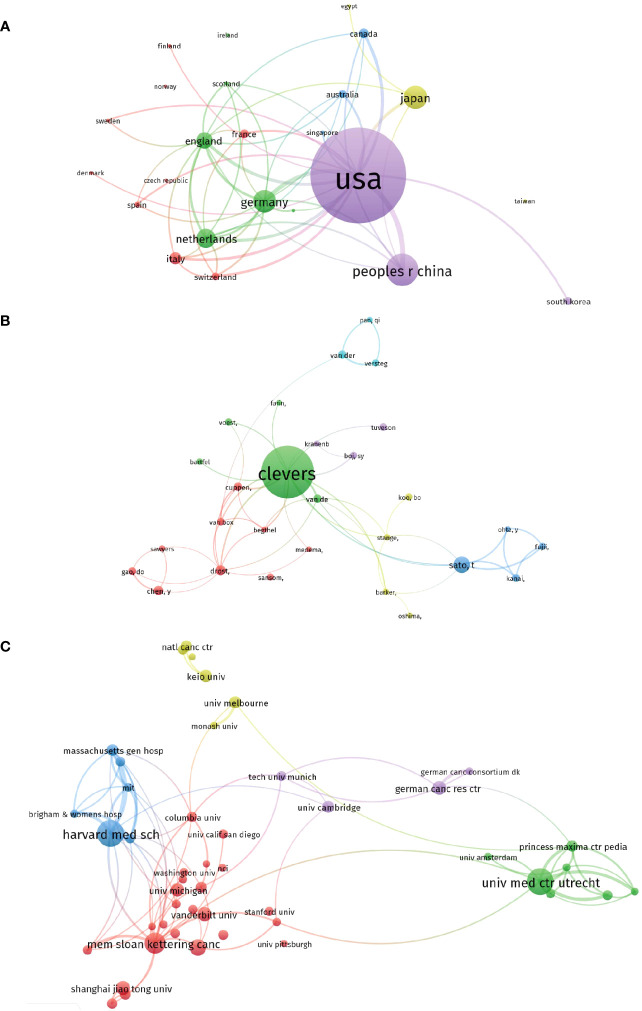
Visualized images of co-authorship analysis of global research about organoids in cancer research. **(A)** Mapping of the 27-country co-authorship analysis on organoids in cancer research. **(B)** Mapping of the 46-author co-authorship analysis on organoids in cancer research. **(C)** Mapping of the 47-institution co-authorship analysis on organoids in cancer research.

In the study, a total of 27 countries that had published more than 20 papers in the field were analyzed using VOS viewer. The analysis aimed to understand the interrelatedness and collaboration patterns between these countries. The findings, presented in **Figure 8A**, revealed the top 5 countries with the highest total link strength: The United States had the highest total link strength of 1009, indicating its strong presence and collaboration in the field of organoid research in cancer. Germany ranked second with a total link strength of 460, suggesting significant contributions and collaborations from German researchers in this area. England followed closely with a total link strength of 399, indicating its active participation and collaboration in organoid research related to cancer. China obtained a total link strength of 337, reflecting its growing presence and involvement in the field, emphasizing the country’s contribution to organoid research in cancer. The Netherlands had a total link strength of 328, demonstrating its strong involvement and collaboration in organoid research, particularly in the context of cancer. These findings provide insights into the leading countries and their collaborative networks in the field of organoid research in the context of cancer.

#### Authors

In the co-authorship analysis using VOS viewer, a total of 46 authors with over 10 documents were analyzed to understand the relatedness and collaboration patterns among them. The analysis aimed to identify the authors with the highest total link strength, indicating their strong co-authorship relationships within the field of organoid research in cancer. The findings, presented in **Figure 8B**, revealed the top 5 authors with the highest total link strength: Hans Clevers had the highest total link strength of 206, indicating his extensive co-authorship relationships with other researchers in the field. Clevers is well-known for his contributions to organoid research, particularly in cancer, and his high link strength reflects his collaborative nature and influence in the field. Toshiro Sato obtained a total link strength of 130, suggesting significant co-authorship relationships and collaborations with other researchers. Sato’s contributions to the field of organoids in cancer research have been influential, and his strong link strength reflects his active involvement in collaborative efforts. Takanori Kanai had a total link strength of 93, indicating his substantial co-authorship relationships within the field. Kanai’s contributions to organoid research, particularly in the context of cancer, have made him a prominent figure in the field and have facilitated collaborations with other researchers. Masayuki Fujii obtained a total link strength of 91, reflecting his strong co-authorship relationships in the field. Fujii’s contributions to organoid research, especially in cancer-related studies, have led to collaborations with other researchers, as evident from his high link strength. Yuki Ohta had a total link strength of 87, highlighting his significant co-authorship relationships within the field. Ohta’s involvement in organoid research, particularly in the context of cancer, has fostered collaborations and partnerships with other researchers. These findings provide insights into the collaborative networks and co-authorship relationships among authors in the field of organoid research in cancer.

#### Institutions

In the analysis conducted using VOS viewer, a total of 47 institutions with more than 30 documents were examined to assess their interrelatedness and collaborations within the field of organoid research in cancer. The aim was to identify the institutions with the highest total link strength, indicating their strong connections and collaborations with other institutions. The findings, presented in **Figure 8C**, revealed the top 5 institutions with the highest total link strength: Harvard Medical School obtained the highest total link strength of 405, indicating its strong connections and collaborations with other institutions in the field. Harvard Medical School is renowned for its contributions to medical research, including organoid research in cancer, and its high link strength reflects its extensive collaborations with other institutions. The University Medical Center Utrecht obtained a total link strength of 334, highlighting its significant connections and collaborations with other institutions. The University Medical Center Utrecht is known for its expertise in medical research, including organoid research in cancer, and its high link strength demonstrates its active engagement in collaborative efforts. The Memorial Sloan Kettering Cancer Center obtained a total link strength of 330, indicating its strong interconnections and collaborations with other institutions. As a leading cancer research center, the Memorial Sloan Kettering Cancer Center plays a crucial role in organoid research and its high link strength reflects its collaborative nature. The German Cancer Research Center obtained a total link strength of 201, highlighting its significant collaborations and connections with other institutions. The German Cancer Research Center is recognized for its contributions to cancer research, including organoid research, and its high link strength reflects its active involvement in collaborative endeavors. The Dana-Farber Cancer Institute obtained a total link strength of 193, indicating its strong interconnections and collaborations with other institutions. The Dana-Farber Cancer Institute is renowned for its research in cancer, including organoid research, and its high link strength underscores its collaborative efforts with other institutions. These findings provide insights into the collaborative networks and connections among institutions in the field of organoid research in cancer, highlighting the institutions with the highest total link strength and their significant collaborations with other institutions.

### Analysis of keywords and hotspots based on co-occurrence analysis

**Figure 9:**
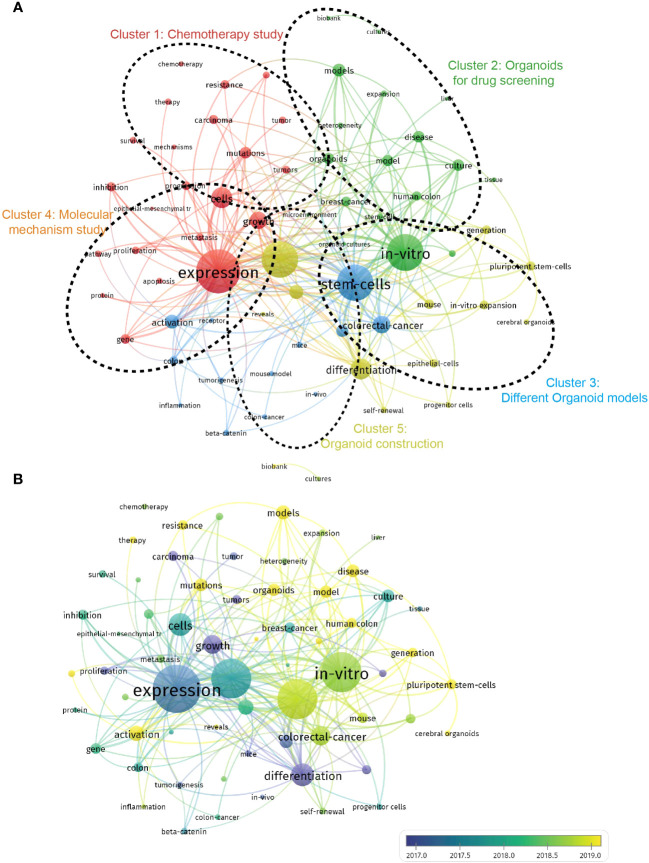
Visualization of co-occurrence analysis based on organoids in cancer research. **(A)** Mapping of keywords in the research on organoids in cancer research; the frequency is represented by point size; The keywords of research fields were divided into five clusters by different colors: chemotherapy study (red), organoids for drug screening (green), different organoid models (blue), molecular mechanism study (orange), organoid construction (yellow). **(B)** Visualization of keywords distribution; The blue color represents an earlier appearance and yellow point appeared later.

In the study’s analysis of keywords using VOS viewer, a total of 65 keywords that appeared more than 50 times in titles or abstracts were examined. The aim was to identify prominent research areas and monitor scientific developments in the field of organoids in cancer research. The results of the analysis, presented in **Figure 9A**, revealed the grouping of these keywords into five clusters:

#### Cluster 1: chemotherapy study (red)

Keywords in this cluster revolved around the study of chemotherapy in relation to organoids in cancer research. Prominent keywords within this cluster included models, precision medicine, and gemcitabine, indicating a focus on developing and utilizing organoid models for studying the efficacy of chemotherapy and exploring precision medicine approaches in cancer treatment.

#### Cluster 2: organoids for drug screening (green)

This cluster focused on the application of organoids in drug screening and discovery. Keywords within this cluster included culture, tissue engineering, and drug discovery, suggesting the use of organoids as valuable tools for testing and evaluating potential drugs, as well as advancements in tissue engineering techniques for organoid development.

#### Cluster 3: different organoid models (blue)

Keywords in this cluster highlighted the diversity and utilization of various organoid models in cancer research. Prominent keywords included in vitro, expansion, and self-renewal, indicating a focus on the in vitro culture and expansion of different types of organoids, as well as their self-renewal capabilities.

#### Cluster 4: molecular mechanism study (orange)

This cluster emphasized the investigation of molecular mechanisms underlying organoids in cancer research. Keywords within this cluster included expression, differentiation, and mutations, suggesting a focus on studying gene expression patterns, cellular differentiation processes, and genetic mutations in organoids to better understand cancer development and progression.

#### Cluster 5: organoid construction (yellow)

Keywords in this cluster centered around the construction and characterization of organoids in the context of cancer research. Frequently used keywords included adenocarcinoma, classification, and organoid differentiation, indicating a focus on developing organoid models that accurately represent specific cancer types, classifying organoids based on their characteristics, and exploring organoid differentiation processes. These clusters represent the most prominent research interests and areas of investigation within the field of organoids in cancer research. By analyzing keyword co-occurrence, researchers can gain insights into trending research topics, monitor scientific developments, and identify the key areas of focus within the field.

**Figure 10:**
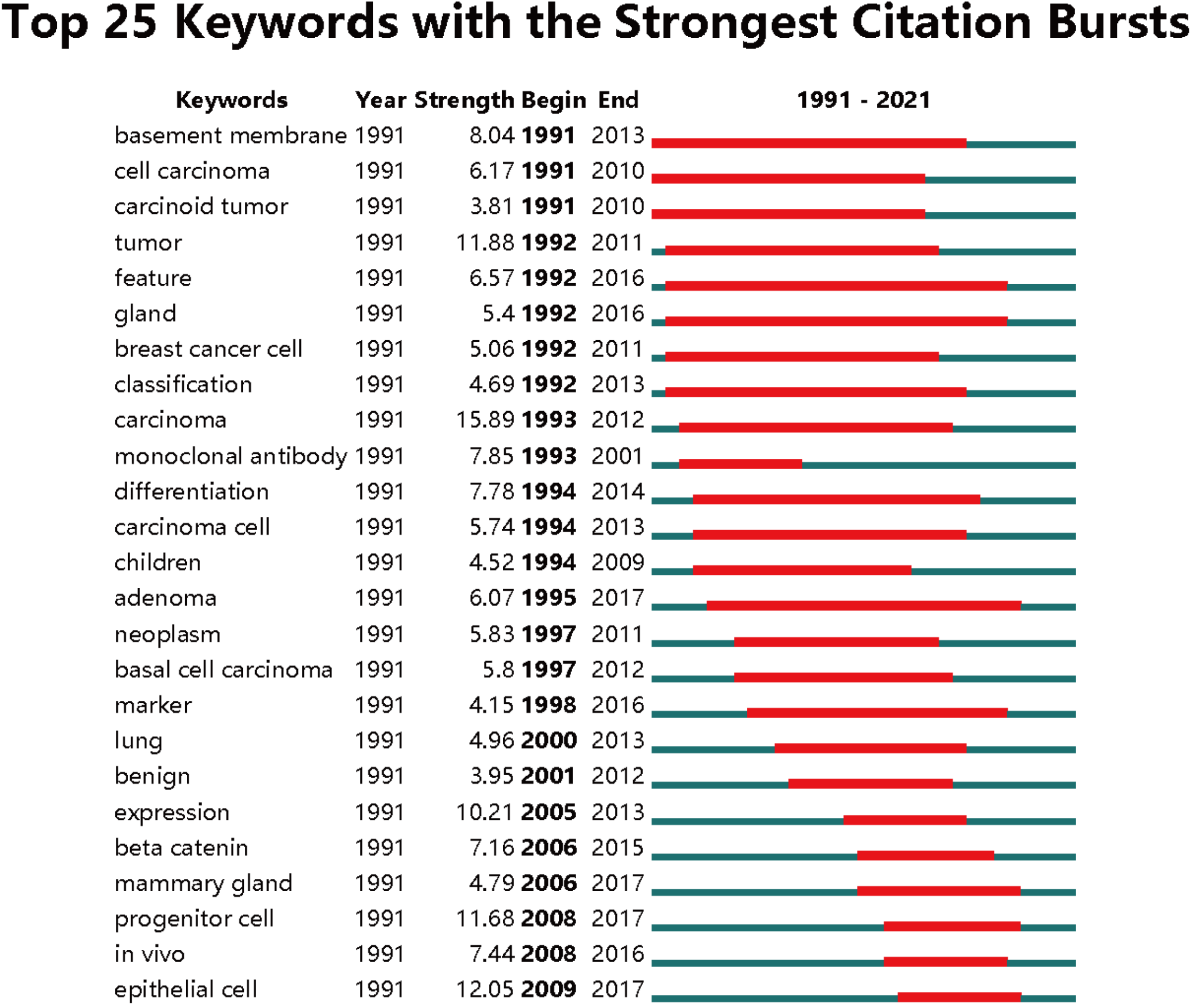
Top 25 keywords with the strongest citation bursts based on CiteSpace.

**Figure 9B** in bibliometrics, which displays color-coded keywords based on their frequency of appearance in published papers, provides valuable insights into the temporal trends of research in the five clusters. The blue-colored keywords indicate an earlier appearance, while the yellow-colored keywords indicate a more recent appearance. The figure reveals that the research trends in the five clusters have remained consistent over time, suggesting that the current research hotspots are likely to continue being the focus in the future. To further analyze the burst strength of keywords, burst detection analysis using CiteSpace was conducted. **Figure 10** presents the top 25 keywords with the highest burst strength. The keyword “carcinoma” exhibited the highest citation outbreaks (strength = 15.89), followed by “epithelial cell” (12.05) and “tumor” (11.88). The keyword “epithelial cell” had the most recent outbreak citations (from 2009 to 2017), indicating that the link between organoids in cancer research and in vitro cell experiments is expected to become a research hotspot in the future. These findings highlight the evolving research landscape in organoids in cancer research and provide insights into the key areas of focus, emerging trends, and the relationships between authors, keywords, and journals.

## Discussion

### Trend of global publications

Cancer research has witnessed remarkable advancements in diagnosis and treatment over the past few decades (6). However, the development of effective treatment regimens remains a significant challenge. In an effort to address this issue, cancer organoids have emerged as a potential alternative to conventional 2D cell line cultures and patient-derived tumor xenografts (PDTXs) for creating tumor models. These 3D self-organized assemblies of neoplastic cells, derived from patient-specific tissue samples, have garnered significant attention in organoid and cancer research (40). In this study, we conducted a comprehensive bibliometric and visual analysis to explore the current research landscape and provide insights into the future development of organoids in cancer research. Our analysis revealed a remarkable increase in the number of publications per year from 1991 to 2021, accompanied by a significant rise in relative research interests (RRIs) in recent years. We identified 73 countries and 2990 institutions that have contributed to the publication of papers in this field. Among them, the United States had the highest number of publications (44.81%), followed by China (15.08%), Japan (11.16%), Germany (10.85%), and the Netherlands (9.09%). Notably, Harvard University, the University of California System, and Utrecht University emerged as the most active contributors to the research front. Our findings underscore the importance of conducting more in-depth studies and fostering collaboration among different institutions and countries to advance organoid development in cancer research. Such collaborations are expected to drive high-quality research and pave the way for significant advancements in the field in the future.

### Quality and status of global publications

Bibliometric analysis provides valuable insights into the impact and productivity of different countries and regions in scientific research. The number of total citations and the H-index are key parameters used to assess the quality and academic impact of countries. As depicted in **Figures 1** and **2**, the USA stands out as the leading country in terms of the number of publications, total citations, and H-index. This highlights the USA’s high productivity and strong research infrastructure, which is in line with its long-standing tradition of scientific research. The USA’s dominant position in this field is not unexpected. However, it is noteworthy that the Netherlands, Austria, and England rank higher than the USA in terms of average citations. This suggests that these countries may have a higher quality of research output, even though they may not match the USA’s level of productivity. The emphasis on quality over quantity in these countries is reflected in their average citation performance. China presents an interesting case in this analysis. While China ranks second in terms of the total number of publications, its total citation count and H-index are relatively lower, ranking fifth and sixth, respectively. This indicates that China has room for significant improvement in terms of the quality of its research output. Despite having numerous elite institutions and researchers, more efforts are needed to enhance the quality of studies in this field. The contrast between the quantity and quality of publications in China emphasizes the importance of the Chinese academic evaluation systems (CAESs) in making greater efforts to improve research quality. In conclusion, bibliometric analysis helps identify the productivity and impact of different countries in scientific research. While the USA leads in terms of productivity, countries like the Netherlands, Austria, and England demonstrate a higher quality of research output. China, with its significant number of publications, can focus on improving the quality of its research to bridge the gap between quantity and impact. In conclusion, bibliometric analysis can provide valuable insights into the productivity and impact of different countries and regions in scientific research. The number of total citations and H-index are critical parameters in bibliometric analysis, and they can help to identify countries with high-quality research output. The USA is currently the most productive country in this field, but the Netherlands, Austria, and England have higher average citations, indicating higher quality research output. China has a high level of productivity but has room for improvement in terms of the quality of research output.

In addition to examining the countries and institutions involved in organoids in cancer research, an analysis of the journals publishing articles in this field is also crucial. **Figure 3** reveals that Cancers, Scientific Reports, and Nature Communications are the top three journals with the highest number of publications in this area. These journals are known for their high impact factors, indicating that the research published in these journals has a significant influence on the academic community. It is interesting to observe that the number of publications in the top two journals, Cancers and Scientific Reports, surpasses the number of articles published in the journals ranked third to fifth. This suggests that these two journals may hold a dominant position in the field of organoids in cancer research. Based on these findings, we predict that the top 25 journals identified in this analysis could continue to be primary channels for publishing high-quality research in the future. Therefore, researchers should consider submitting their work to these journals to maximize its impact and visibility within the scientific community.

The analysis of institutions is essential for evaluating the contributions of different research institutes to the field of organoids in cancer research. Our study demonstrates that the leading institutes from the top 5 countries have made significant contributions in this field, aligning with the global publications produced by these countries. It is noteworthy that all of the top 25 institutes identified in this analysis are from these top 5 countries, underscoring the pivotal role played by top-tier research institutions in elevating a country’s academic ranking. By considering the countries, institutions, and journals highlighted in this analysis, researchers can gain insights into the most productive and influential entities in the field of organoids in cancer research. This knowledge can guide researchers in selecting collaboration partners, identifying potential publication outlets, and strategically positioning their work for maximum impact and recognition.

Furthermore, our study also identified the top-ranked authors who made significant contributions to the field of organoids in cancer research. Many of these authors were affiliated with institutions in the United States, with Harvard University being the top-ranked institution. It is noteworthy that the United States Department of Health and Human Services provided substantial funding for this research, further emphasizing the influential role of the United States in driving advancements in this field. The top-ranked authors listed in **Figure 4B** are considered early pioneers in this field and likely possess valuable insights into the latest advancements in organoids in cancer research.

In addition to identifying leading authors and institutions, bibliometric research methods can also be employed to identify hotspots in previous reports. Our study utilized coupling analysis to establish connections among articles based on institutions, journals, countries, and authors. The results revealed that Cancers was the most closely related journal, Harvard University was the most closely related institution, the United States was the most closely related country, and Clevers, Hans was the most closely related author in the field of organoids in cancer research. These findings serve as valuable resources for researchers seeking to identify key players and emerging trends in this field and can guide future research directions. By leveraging bibliometric analysis, researchers can gain a comprehensive understanding of the prominent authors, institutions, journals, and countries shaping the field of organoids in cancer research. This knowledge can inform collaborations, highlight influential research outlets, and facilitate the identification of emerging research areas, ultimately advancing the progress and impact of organoid research in the context of cancer.

In our study, we conducted a co-citation analysis based on journals and references to assess the impact of publications by examining the total number of citations they received. The results, presented in **Figure 6**, highlighted the influential studies in the field of organoids in cancer research, which received the highest citation frequencies. These landmark studies have made significant contributions to advancing the understanding and development of organoids in the context of cancer. Furthermore, co-creational analysis of journals allowed us to identify the journals that have made outstanding contributions to this field. It is likely that Nature, based on the highest citation frequency, emerged as the top journal with a significant impact on organoid research in cancer. The recognition and citation of studies published in these influential journals serve as an indication of their valuable contributions to the field.

Co-authorship analysis provided insights into the connections between authors, institutions, and countries. By evaluating the total link strength, we can identify authors, institutions, and countries that exhibit strong collaborative relationships. It is crucial for authors, institutions, and countries with higher total link strength to foster collaborative efforts and work together more closely. This cross-cooperation and collaboration can enhance communication, productivity, and ultimately improve the research level in the specific subject of organoids in cancer research. Based on these findings, we provided perspectives and suggestions that emphasize the importance of cross-cooperation in future research endeavors. Encouraging collaborative efforts among authors, institutions, and countries can facilitate knowledge sharing, interdisciplinary approaches, and the generation of high-quality research outcomes. By promoting communication and productivity through collaboration, the overall research level in the field of organoids in cancer research can be further improved.

### Research focus on organoid in cancer research

The keyword co-occurrence analysis conducted in this study revealed the emerging trends and hotspots in organoids in cancer research. By analyzing the occurrence network of keywords in the titles and abstracts of the included documents, we identified five main research trends, as presented in **Figure 8A**. These trends not only align with the critical hotspots in the field but also provide insights into the future directions of investigation. Let’s explore these trends in more detail:

Chemotherapy study: This trend emphasizes the importance of investigating the effectiveness of chemotherapy treatments using organoids as a model. Researchers can explore how organoids respond to different chemotherapy drugs, identify mechanisms of drug resistance, and optimize drug dosages using organoids. This line of research can contribute to improving the efficacy of chemotherapy in cancer treatment.

Organoids for drug screening: The second trend highlights the promising application of organoid technology in drug screening. Organoids can be used as powerful tools to screen large numbers of drugs in a high-throughput manner, enabling more efficient and cost-effective drug discovery processes. By using organoids for drug screening, researchers can identify potential candidates for further development and study. Different organoid models: This trend indicates that researchers are exploring the use of different types of organoids in cancer research. For instance, patient-derived organoids (PDOs) are gaining attention as they provide a more relevant and personalized model for studying individual patients’ tumors. By incorporating various organoid models, researchers aim to improve the relevance and accuracy of their experimental models, ultimately enhancing the translational potential of organoid research.

Molecular mechanism study: Understanding the molecular mechanisms that underlie cancer development and progression is crucial for advancing cancer research. Organoids offer a physiologically relevant context to study these molecular mechanisms. By using organoids, researchers can investigate gene expression patterns, cellular differentiation processes, mutations, and other molecular events that contribute to cancer progression. This trend highlights the significance of studying molecular mechanisms using organoid models.

Organoid construction: The final trend emphasizes the ongoing efforts to improve and optimize organoid culture methods. This includes the development of new scaffolds, culture media formulations, and other techniques to enhance organoid growth, functionality, and maturation. By refining organoid construction techniques, researchers aim to create more reliable and reproducible organoid models that better mimic the complex biology of tumors, thus enhancing their utility in cancer research. These trends provide valuable insights into the current research landscape of organoids in cancer research and offer guidance for future investigations. By focusing on these research areas, researchers can contribute to advancing our understanding of cancer biology, improving drug discovery processes, and developing more effective therapeutic strategies.

(I). The field of chemotherapy study in organoids for cancer research has identified several key future directions through the co-occurrence analysis of keywords. One important area is the application of precision medicine in chemotherapy, aiming to differentiate patients who will respond well to chemotherapy from those who will not. To achieve this goal, predictive biomarker assays can be developed to guide chemotherapy treatment decisions, and patient-derived cancer organoids can serve as a valuable platform for the development of such assays.

The analysis highlighted the keywords “models,” “precision medicine,” and “gemcitabine” as crucial areas for future research. Traditionally, chemotherapy has not been considered a precision medicine because of the diverse responses observed among patients. However, it is increasingly recognized that predictive biomarker assays could enhance the effectiveness of chemotherapy (41).

For instance, a clinical study with metastatic colorectal cancer (mCRC) patients investigated the response to standard-of-care chemotherapy using patient-derived tumor organoids. The researchers treated 35 tumor organoid lines with a combination of fluorouracil (5-FU) and oxaliplatin (FO) or irinotecan, or irinotecan alone. They found a correlation between the ex vivo treatment response of the organoids and the patients’ clinical response to irinotecan monotherapy, but not with oxaliplatin-based treatment (42).

However, it is important to note that these studies have limitations, such as the lack of a strong correlation between in vitro chemotherapeutic results and drug responses observed in patients. Therefore, further prospective cancer organoid studies are needed to provide a more comprehensive understanding of the potential benefits of precise chemotherapy. Overall, the application of precision medicine in chemotherapy using patient-derived cancer organoids holds significant potential to improve treatment outcomes and warrants further research.

(II). Organoids for drug screening have emerged as a crucial field in cancer research, and the use of patient-derived organoids provides a valuable platform for studying drug response in vitro. Organoids have been successfully employed to assess drug response in various cancer types, such as breast cancer, ovarian cancer, and colorectal cancer. For instance, Chaudhuri et al. utilized matched samples of a PARP-inhibitor-sensitive and -resistant BRCA2-mutated mammary tumor to measure drug response in breast tumor organoids (43). This approach has the potential to identify new drug targets and drug combinations for cancer treatment.

Moreover, the essentialome of an individual tumor can be determined using a rapid and robust genome-wide CRISPR/Cas9 mutagenesis assay (44). This technique helps identify specific vulnerabilities of a particular tumor and guides personalized treatment for the patient. The organoid platform is also instrumental in testing new drugs and drug combinations, leading to the development of more effective cancer treatments. Additionally, the “sensitive-to-resistant essentialome” comparison approach can be employed, involving the acquisition and screening of new organoid lines when a tumor relapses or becomes resistant to treatment. This approach can provide valuable insights into treatment options and uncover new information about vulnerabilities for clinical patients.

Overall, organoids for drug screening is a promising field that has the potential to improve cancer treatment by identifying specific drug targets and drug combinations for personalized therapy. By utilizing patient-derived organoids, researchers can gain a better understanding of drug response and develop more effective treatment strategies for individual patients.

(III). In recent years, there has been a growing emphasis on developing different organoid models to enhance the representation of tumor complexity and its microenvironment. Personalized tumor models that accurately capture the heterogeneity of individual patients are crucial for advancing precision medicine. Researchers worldwide have generated various epithelial cancer organoid cultures, including colon, liver, pancreas, prostate, stomach, lung, and breast organoids (13–19).

Angiogenesis, which is essential for tumor growth and metastasis, has also been a focus of research in organoid development (45). Constructing vascularized tumoroids that better mimic in vivo tumor conditions has been an area of interest. One approach involves coculturing tumor cells with lung fibroblasts and endothelial cell-derived endothelial colony-forming cells to create vascularized tumoroids (46). This approach aims to incorporate the vascular component into the organoid model, enabling better representation of the tumor microenvironment.

Another significant research direction is the investigation of the interplay between tumors and immune cells. Tumor organoids, in combination with autologous immune cells, can provide a valuable platform for modeling immune system function in tumors (47). For example, Kuen et al. developed a 3D coculture system by coculturing pancreatic cancer cells with monocytes and cancer-associated fibroblasts, which successfully induced increased secretion of immunosuppressive cytokines in vitro (48). Such advancements in organoid technology, particularly through coculture systems, hold promise for creating an in vitro platform to analyze tumor response from both adaptive and innate immune perspectives.

In summary, the development of diverse organoid models, including vascularized tumoroids and immune cell-inclusive organoids, aims to improve the representation of tumor complexity and microenvironment in vitro. These advancements in organoid technology offer new avenues for studying tumor biology, drug responses, and the interplay between tumors and immune cells, ultimately contributing to the advancement of precision medicine and personalized cancer therapy.

(IV). The construction and study of cancer organoids provide valuable insights into the molecular mechanisms underlying cancer development and progression. By better recapitulating the 3D architecture and cellular heterogeneity of tumors, organoids offer a more physiologically relevant model compared to traditional 2D cell lines and patient-derived tumor xenografts. This enhanced model enables researchers to investigate cancer biology and drug response in a more realistic and informative manner. One active area of research in cancer organoids is the exploration of infectious agents and their role in cancer development. Organoids can be infected with various pathogens to investigate how these infections impact tumor growth and progression. For example, the study by Scanu et al. demonstrated that Salmonella enterica infection of gallbladder organoids with TP53 mutations and MYC amplifications activates AKT and MAPK signaling, thereby promoting neoplastic transformation (49). Such investigations can contribute to the identification of new therapeutic targets and the development of novel treatments for infectious agent-related cancers.

Additionally, cancer organoid research focuses on studying the effects of genetic mutations on tumor development and drug response. Mutations play a crucial role in tissue homeostasis and tumorigenesis, and understanding specific mutations within tumors can guide personalized treatment strategies. For instance, studies have revealed that tumor cells lacking BRCA1 or BRCA2 genes exhibit sensitivity to PARP inhibitors, suggesting a potential treatment option for patients with these mutations (50). The utilization of cancer organoids to investigate the molecular mechanisms underlying drug response can aid in identifying new treatment opportunities and improving patient outcomes. In summary, cancer organoids serve as an advanced model for studying the molecular mechanisms involved in cancer pathogenesis, the effects of infectious agents, and the influence of genetic mutations on tumor development and drug response. These investigations enhance our understanding of cancer biology, facilitate the development of targeted therapies, and contribute to the advancement of precision medicine approaches in cancer treatment.

In conclusion, the use of cancer organoids as a research platform holds great promise for gaining a deeper understanding of the molecular mechanisms of cancer development and drug response. As more researchers adopt this technology and explore new methods for generating and studying organoids, we can expect to make significant strides in our knowledge of cancer biology and develop more effective treatments for this devastating disease.

(V). Organoid generation is a critical aspect of organoid culture, and various substrates and materials are utilized to create a suitable environment for the growth and differentiation of organoids. Matrigel, a basement membrane matrix derived from mouse sarcoma cells, has been widely used as a substrate due to its compatibility with organoid culture. However, researchers are actively exploring alternative materials to overcome limitations associated with Matrigel. Natural polymer-based hydrogels, such as gelatin, fibrin, collagen, silk, and tissue extracellular matrix (ECM), are favored for their resemblance to the ECM and their biological activity. These materials provide a more physiologically relevant environment for organoid culture. For example, collagen-based scaffolds can induce distinct resistance mechanisms and pathological features depending on the cell type, making them a suitable alternative to Matrigel.

Synthetic hydrogels, such as polyethylene glycol (PEG) and its derivatives, poly (D,L-lactic acid), polycaprolactone (PCL), and poly (Ethylene Oxide)/poly (Butylene Terephthalate) (PEOT/PBT), are also being explored as substitutes for Matrigel. These synthetic materials offer high versatility, reproducibility, and favorable biophysical properties for organoid culture. Hybrid hydrogels, created through physical blends and chemical copolymerization/modification, enhance functionality and better simulate the dynamic microenvironment in vivo.

The development and application of organoid culture media are also crucial for long-term expansion of organoids and the modeling of specific developmental processes and diseases. Advancements in culture media formulation are anticipated to further improve the growth and functionality of organoids. In recent years, two research topics that have gained significant attention in the field of organoids in cancer research are “organoid differentiation” and “adenocarcinoma.” The differentiation of organoids aims to mimic the complex cellular organization and functionality of native tissues. Researchers are exploring various strategies and culture conditions to promote the differentiation of organoids into specific cell types. In the context of adenocarcinoma, studies are focused on modeling and studying this specific type of cancer using organoids. The goal is to better understand the disease and develop targeted therapies.

An example study by Below et al. demonstrated the development of a fully synthetic hydrogel that mimics the pancreatic ECM environment, specifically targeting laminin-integrin α3/α6 signaling. This research highlighted the importance of the ECM in promoting the survival and differentiation of pancreatic organoids. While significant progress has been made in organoid construction research, there is still ample room for further advancements. Ongoing research in this field has the potential to greatly contribute to our understanding of cancer biology and the development of effective cancer treatments. The co-occurrence map and clusters identified in the study provide insights into the general trends and research directions in the field. These clusters represent potential areas of interest for future research, as indicated by their associated scores. By exploring and addressing the challenges and opportunities identified through via co-occurrence analysis, researchers can further advance the field of organoids in cancer research.

### Research future trends

The increasing interest in organoids for cancer research has indeed sparked predictions about future research directions and potential impact. **Figure 10** highlights several key areas that are likely to shape the future of organoid research in cancer. Cancer organoid culture: The construction of cancer organoids has already shown great promise. However, commercial cell lines cultured on conventional monolayer supports are in vitro systems not able to fully mimic the microenvironment of cancer diseases. 3D models, organ-on-chip or tissue-derived cultures represent valuable research tools able to increase the reliability of in vitro and in vivo systems through the integrations of different models (51–53). Future research is expected to focus on refining and optimizing organoid culture techniques, for example, integrating different systems, to better mimic the complexity of tumors. This includes developing methods to generate organoids from various stem cell populations and improving the differentiation and maturation of organoids. Organoid techniques for cancer biology research: Researchers are interested in leveraging organoids to gain insights into cancer biology. This involves using organoids to study tumor development, organogenesis, and the interactions between cancer cells and the immune system. CRISPR-based genetic modifications can be employed to introduce specific oncogenes or study the effects of genetic alterations on tumor progression. Tumor organoid biobanks: The development of tumor organoid biobanks is a promising direction for future research. These biobanks involve the collection and preservation of organoids derived from different types of cancers, with varying grades and stages. This resource can enable personalized medicine approaches, such as drug screening and testing, and facilitate the study of tumor heterogeneity and response to therapy. The potential impact of these advancements is significant. Cancer organoids have the potential to provide more accurate and physiologically relevant models for studying cancer biology, drug response, and personalized medicine. The establishment of tumor organoid biobanks can facilitate translational research and improve clinical outcomes by enabling the development of tailored treatment strategies.

In conclusion, the ongoing advances in organoid technology and the allocation of research funding are expected to drive significant progress in cancer research. The future of organoid research in cancer will likely revolve around cancer organoid culture, organoid techniques for facilitating cancer biology research, and the establishment of tumor organoid biobanks. These areas hold tremendous potential for advancing our understanding of cancer and improving patient care.

### Limitation

The acknowledgement of limitations is an important aspect of any research study. Indeed, there are a few limitations to consider regarding the analysis conducted using the WoS database for the insights into organoids in cancer research. Database selection: The study focused solely on the WoS database, which might not cover all relevant publications in the field. Other databases, such as PubMed, Cochrane, and Embase, provide access to a wider range of literature and could contain additional relevant studies. Including these databases in future research would offer a more comprehensive analysis. Language bias: The study only considered publications in English, which could result in the exclusion of valuable contributions from non-English language publications. This language bias may limit the representation of certain research findings and perspectives. Including publications in different languages can provide a more inclusive overview of global trends and interests. Citation-based analysis: The analysis relied on the citation counts of publications as a measure of their impact and influence. While citations can indicate the level of recognition within the scientific community, they may not capture the most recent or cutting-edge research. Newer high-quality papers may not have accumulated enough citations at the time of analysis, potentially leading to a discrepancy between the bibliometric analysis and the current state of research. To address these limitations, future research can consider a more comprehensive approach by incorporating multiple databases and including publications in different languages. This would provide a broader view of the research landscape and reduce the potential for bias. Additionally, combining bibliometric analysis with other methods, such as expert surveys or qualitative assessments, can offer a more comprehensive understanding of trends and interests in organoids in cancer research.

## Conclusion

Our bibliometric analysis study reveals a remarkable global interest in the field of organoid and cancer research spanning the period from 1991 to 2021. The findings indicate that the United States takes the lead in terms of publications pertaining to organoids in cancer research, demonstrating its significant contribution and influence in this area. This is further supported by the highest total citation frequencies and H-index, highlighting the country’s prominent role. Notably, the journal Cancers has emerged as the primary platform for publishing on this topic, indicating its significance as a source of scientific discourse. The study also predicts a continued growth in the number of publications on organoids in cancer research in the future, reflecting the sustained interest and ongoing advancements in the field. However, despite the increasing attention, it is evident that organoids in cancer research still do not receive sufficient global recognition. Further efforts are needed to raise awareness and promote wider engagement with this important area of study. Moving forward, it is anticipated that future research directions will center around several key areas, including the investigation of chemotherapy, the application of organoids for drug screening, the development of diverse organoid models, the study of molecular mechanisms, and the refinement of organoid construction techniques. These research foci align with the current trends and emerging opportunities in the field, aiming to enhance our understanding of cancer and improve therapeutic strategies. Nonetheless, it is essential to acknowledge the limitations of our study. The exclusion of major databases such as PubMed, Cochrane, and Embase library databases may have impacted the comprehensiveness of our data, potentially omitting valuable contributions. Moreover, the presence of language bias could have led to the inadvertent exclusion of non-English publications, limiting the global perspective of our analysis. Additionally, it is important to recognize that bibliometric analysis, reliant on citation metrics, may not entirely reflect the real-world research landscape, particularly when considering emerging and less-cited studies. To provide more nuanced insights into organoids in cancer research, further analysis incorporating a broader range of databases, languages, and research methodologies is warranted.

## Data availability statement

The original contributions presented in the study are included in the article/supplementary material. Further inquiries can be directed to the corresponding author.

## Author contributions

ST: Conceptualization, Data curation, Formal Analysis, Investigation, Methodology, Software, Visualization, Writing – original draft. JD: Conceptualization, Data curation, Formal Analysis, Writing – original draft. HD: Conceptualization, Data curation, Formal Analysis, Investigation, Writing – original draft. LL: Conceptualization, Data curation, Project administration, Writing – original draft. ZQ: Resources, Software, Validation, Visualization, Writing – original draft. YL: Conceptualization, Software, Supervision, Writing – original draft. LT: Conceptualization, Investigation, Project administration, Writing – review & editing. ZL: Funding acquisition, Resources, Software, Validation, Writing – review & editing.
